# A Sanger sequencing protocol for SARS‐CoV‐2 S‐gene

**DOI:** 10.1111/irv.12892

**Published:** 2021-08-03

**Authors:** Rodney S. Daniels, Ruth Harvey, Burcu Ermetal, Zheng Xiang, Monica Galiano, Lorin Adams, John W. McCauley

**Affiliations:** ^1^ Worldwide Influenza Centre (WIC: a WHO Collaborating Centre for Reference and Research on Influenza) The Francis Crick Institute London UK

**Keywords:** base‐calling accuracy, Sanger sequencing, SARS‐CoV‐2, S‐gene, variant detection

## Abstract

We describe a Sanger sequencing protocol for SARS‐CoV‐2 S‐gene the Spike (S)‐glycoprotein product of which, composed of receptor‐binding (S1) and membrane fusion (S2) segments, is the target of vaccines used to combat COVID‐19. The protocol can be used in laboratories with basic Sanger sequencing capabilities and allows rapid “at source” screening for SARS‐CoV‐2 variants, notably those of concern. The protocol has been applied for surveillance, with clinical specimens collected in either nucleic acid preservation lysis‐mix or virus transport medium, and research involving cultured viruses, and can yield data of public health importance in a timely manner.

## 1 INTRODUCTION

In many countries, National Influenza Centres (NICs), which form the backbone of the World Health Organization (WHO) Global Influenza Surveillance and Response System (GISRS), have become major centres for SARS‐CoV‐2 surveillance. WHO has published operational considerations for COVID‐19 surveillance using GISRS^1^ and for preparation of NICs for upcoming influenza seasons^2^ with SARS‐CoV‐2 having displaced seasonal influenza since the end of March 2020.

Gene sequencing is key for surveillance of SARS‐CoV‐2 and monitoring for the emergence of mutated strains of the virus which may have altered behaviour and infectivity/transmissibility characteristics that affect spread of the disease and/or disease severity,^3^, ^4^ together with the capacity to potentially escape protective immunity induced by vaccination (thereby reducing vaccine efficacy) and/or previous infection,^5^ and/or escape methods of virus detection such as real‐time RT‐PCR (rtRT‐PCR) assays. Infectivity/transmissibility characteristics and immune evasion are particularly relevant to the S‐gene that encodes the surface S‐glycoprotein (Spike) that is responsible for initiating infection by binding to host cell ACE2 receptor,^6^, ^7^ fusion of virus and cell membranes, and release of the virus genome, which then uses cellular machinery to produce progeny virus that disseminates within the host and fuels virus transmission to new hosts. These activities, together with Spike being the major inducer of host neutralising antibody responses, make it a target for therapeutic strategies and vaccine development.^8^, ^9^

In this age of next generation sequencing (NGS) methodologies, the great majority of protocols developed (e.g., ARTIC [https://artic.network/ncov-2019] where a number of relatively short [~400 bp] genome fragments are generated to cover the entire genome [~30 000 nucleotides]) are focussed on whole genome sequencing. The adoption of such protocols by many sequencing laboratories has resulted in the deposition of 1 563 032 SARS‐CoV‐2 virus sequences (as of 2021‐05‐15) in the Global Initiative on Sharing All Influenza Data (GISAID) EpiCoV™ database. However, numbers are reduced significantly when a combination of search categories are selected consecutively: “complete” (n = 1 532 515) and “high coverage” (n = 1 177 811), which relate to sequence quality, and “collection date complete” (n = 1 151 800), which is an important criterion for data analyses. The latter number represents a 26% reduction in the number of “quality” sequences. Further, many of these “quality” sequences contain significant runs of four or more undefined/missing nucleotides (n: see below for a focus on the S‐gene) that cause major issues for alignment programmes such as MAFFT (https://mafft.cbrc.jp/alignment/software/) resulting in alignments with sizeable gaps, often encompassing coding regions of important S‐glycoprotein domains (e.g., the receptor‐binding domain and associated antigenic sites, and the S1/S2 cleavage site). In developing the set of 16 primers reported here for a S‐gene specific Sanger sequencing approach, 10 823 “quality” sequences from the initial stages of the COVID‐19 pandemic were downloaded from the EpiCoV™ database on 2020‐04‐29 and a MAFFT‐generated alignment made, from which the S‐gene coding section (with some flanking sequence) was extracted. Having extracted the S‐gene coding region sequences with runs of four or more n were removed, leaving 8 429 (an additional 22% reduction), which were re‐aligned. Further, many of these remaining sequences contained significant numbers of nucleotide ambiguity codes, often occurring in runs, that possibly relate to the base‐calling capabilities of the NGS platform and the quality of the bioinformatics pipeline used,^10^, ^11^ together with amount and quality of SARS‐CoV‐2 RNA recovered from clinical specimens.

Sanger‐based sequencing protocols for the S‐gene are available, for example, a commercially available set of 24 M13‐tagged primers^12^ linked to use of specified equipment (https://assets.thermofisher.com/TFS-Assets/GSD/brochures/sequencing-sars-cov-2-spike-gene-protocol.pdf). However, a significant number of NICs within GISRS in low‐middle income countries (LMICs) do not have the resources or within country support to either upgrade their existing Sanger sequencing facilities or implement and maintain NGS in a cost effective manner (which is dependent on a high throughput of samples). The Sanger sequencing approach described here has been shared with a small number of NICs where it has been implemented successfully based on their existing methods and capabilities that have been developed for influenza surveillance.

Table 1 gives details of the primer set together with primer pairings used to produce three overlapping fragments (each of ~1700 bp) that cover the S‐gene, primer sets used to sequence the individual fragments and conditions used for RT‐PCR generation of the fragments. All primers (1‐μmol scale) were ordered from Sigma‐Aldrich (now Merck) with HPLC purification and supplied in water @ 100 μM. The protocol has been and continues to be used successfully with SARS‐CoV‐2 positive clinical specimens, generally working well for specimens with rtRT‐PCR Ct values up to 20 (intermittently with those having values up to 30) and cultured viruses covering the range of “Variants of Concern” (VoC) identified to date (https://www.ecdc.europa.eu/en/covid-19/variants-concern), indicating the robustness of the method. Specifically, it has been used effectively with the Wuhan strain, viruses of the first wave in England and a wide range of VoC as of 2021‐05‐15, notably B.1.117 (Kent/Alpha), B.1.351 (South Africa/Beta), P.1 (Brazil/Gamma), and B.1.617.2 (India/Delta). It is being used routinely to screen for potential Spike amino acid substitutions and/or polymorphisms that may emerge during adaptation of SARS‐CoV‐2 to propagation in cell‐lines used in the laboratory, and validating of virus stocks generated for use in high throughput assays, for example, virus neutralisation assays used to screen for potential escape of new variants from antibody responses induced by vaccination.^5^, ^13^, ^14^ In addition, NGS has been performed by mixing the three fragments, performing library preparation with QIAGEN QIAseq FX DNA Library Kits (#180475) and running products on Illumina MiSeq platforms, allowing greater in depth assessment of the presence of minority variants than is available through Sanger sequencing alone.

**TABLE 1:**
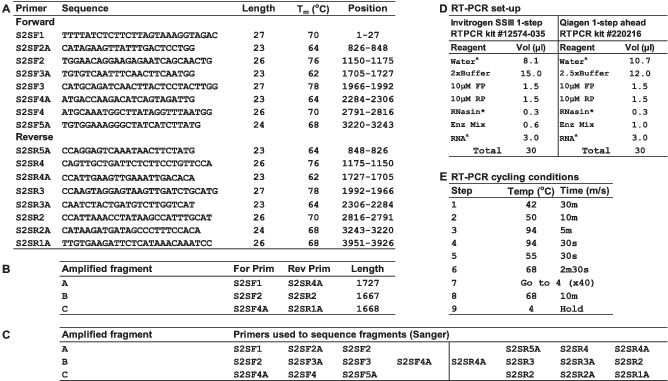
Sanger sequencing of SARS‐CoV‐2 Spike genes: primers used and RT‐PCR conditions

*Not* *e*: (A) Primer name, sequence, length, hybridisation temperature (T_m_), and location across the SARS‐CoV‐2 genome fragment spanning the Spike glycoprotein open‐reading frame (ORF) are shown. Primers S2SF1 and S2SR1A are positioned in highly conserved regions that flank the S‐gene. Based on the primer position numbers indicated in panel A, residues 94–96 would represent the ATG start codon of the S‐gene. (B) The Spike ORF was amplified in three overlapping fragments using the primers indicated. (C) Sanger sequencing of individual fragments used the primer sets indicated. (D) RT‐PCR set‐up is shown for 1‐step kits supplied by Invitrogen or Qiagen, standard reaction set‐up is shown but ^a^water and ^a^RNA volumes can be adjusted to allow addition of more RNA from specimens yielding rtRTPCR Ct values of ≥25. * Promega RNasin® ribonuclease inhibitor (#N2515). (E) Thermal cycling conditions used on a Bio‐Rad DNA ENGINE DYAD Peltier thermal cycler are shown, with temperatures being calculated.

## 2 CONCLUSION

Sanger sequencing remains the “gold standard” for accuracy of base‐calling and thereby quality of sequences generated for surveillance and research purposes. Accuracy of sequencing is essential to prevent databases being flooded with poorly curated sequences and consequent difficulties, and potential erroneous outcomes, when applying bioinformatic softwares to mine the data. Further, gene sequencing alone cannot provide all the information required to fully understand virus evolution and make truly informed vaccine strain selections: phenotypic characterisation of viruses remains paramount when making such decisions. A Spike‐specific Sanger sequencing approach, like that described here, can facilitate rapid identification of clinical specimens containing variants of concern/interest/high consequence (https://www.cdc.gov/coronavirus/2019-ncov/variants/variant-info.html) from which it would be advantageous to recover SARS‐CoV‐2 isolates. To enable this, clinical specimens should be collected in a way that preserves virus viability (use of a suitable virus transport medium, maintenance of cold‐chains during transport and storage at −70°C or below) to allow isolation attempts in laboratories with the necessary biological containment (currently BSL3 for SARS‐CoV‐2) and suitably trained personnel. Before attempting either gene sequencing or virus isolation, particularly in resource limited situations, diagnostic rtRT‐PCR Ct values should be used as a guide to probability of success.

## AUTHOR CONTRIBUTIONS

**Rod Daniels:** Conceptualization; data curation; formal analysis; funding acquisition; investigation; methodology; project administration; resources; software; supervision; validation; visualization. **Ruth Harvey:** Investigation; methodology; project administration; resources. **Burcu Ermetal:** Data curation; formal analysis; investigation; methodology; software; visualization. **Zheng Xiang:** Data curation; formal analysis; methodology; resources; software; validation. **Monica Galiano:** Data curation; formal analysis; investigation; methodology; validation. **Lorin Adams:** Data curation; formal analysis; methodology; software. **John McCauley:** Funding acquisition; project administration; resources; supervision.

## CONFLICT OF INTEREST

All authors declare no conflicts of interest.

## ETHICAL STATEMENT

The conception and execution of this work did not require ethical approval.

## PATIENT CONSENT

All clinical specimens were collected with consent to be used for diagnostic and virus characterisation purposes.

### PEER REVIEW

The peer review history for this article is available at https://publons.com/publon/10.1111/irv.12892.

## Data Availability

Sequence data have been provided to the sharers of clinical specimens for their perusal and deposition in the EpiCoV™ database of GISAID.
